# Cost analysis in a randomized trial of early closure of a temporary ileostomy after rectal resection for cancer (EASY trial)

**DOI:** 10.1007/s00464-019-06732-y

**Published:** 2019-03-25

**Authors:** Jennifer Park, Eva Angenete, David Bock, Adiela Correa-Marinez, Anne K. Danielsen, Jacob Gehrman, Eva Haglind, Jens E. Jansen, Stefan Skullman, Anette Wedin, Jacob Rosenberg

**Affiliations:** 1 Department of Surgery, Institute of Clinical Sciences, Sahlgrenska Academy, Scandinavian Surgical Outcomes Research Group, University of Gothenburg, Sahlgrenska University Hospital, Östra, Gothenburg, 416 85 Sweden; 2 Department of Gastroenterology, Herlev and Gentofte Hospital, University of Copenhagen, Herlev, Denmark; 3 Department of Surgery, Nordsjaellands Hospital, Hillerød, Denmark; 4 Department of Surgery, Skaraborgs Sjukhus, Skövde, Sweden; 5 Department of Surgery, Centre for Perioperative Optimization, Herlev and Gentofte Hospital, University of Copenhagen, Herlev, Denmark

**Keywords:** Rectal cancer, Temporary ileostomy, Rectal resection, Cost analysis

## Abstract

**Background:**

Hospital costs associated with the treatment of rectal cancer are considerable and the formation of a temporary stoma accounts for additional costs. Results from the EASY trial showed that early closure of a temporary ileostomy was associated with significantly fewer postoperative complications but no difference in health-related quality of life up to 12 months after rectal resection. The aim of the present study was to perform a cost analysis within the framework of the EASY trial.

**Methods:**

Early closure (8–13 days) of a temporary stoma was compared to late closure (> 12 weeks) in the randomized controlled trial EASY (NCT01287637). The study period and follow-up was 12 months after rectal resection. Inclusion of participants was made after index surgery. Exclusion criteria were diabetes mellitus, steroid treatment, signs of postoperative complications or anastomotic leakage. Clinical effectiveness and resource use were derived from the trial and unit costs from Swedish sources. Costs were calculated for the year 2016 and analysed from the perspective of the healthcare sector.

**Results:**

Fifty-five patients underwent early closure, and 57 late closure in eight Swedish and Danish hospitals between 2011 and 2014. The difference in mean cost per patient was 4060 US dollar (95% confidence interval 1121; 6999, *p* value < 0.01) in favour of early closure. A sensitivity analysis, taking protocol-driven examinations into account, resulted in an overall difference in mean cost per patient of $3608, in favour of early closure (95% confidence interval 668; 6549, *p* value 0.02). The predominant cost factors were reoperations, readmissions and endoscopic examinations.

**Conclusions:**

The significant cost reduction in this study, together with results of safety and efficacy from the randomized controlled trial, supports the routine use of early closure of a temporary ileostomy after rectal resection for cancer in selected patients without signs of anastomotic leakage.

**Clinical trial:**

Registered at clinicaltrials.gov, clinical trials identifier NCT01287637.

Surgical treatment of potentially curable rectal cancer includes low anterior resection (LAR) with total mesorectal excision (TME) [1, 2]. Because of the proximity to the pelvic floor and based on previous results, the routine procedure involves the formation of a temporary defunctioning stoma in order to reduce the risk of symptomatic anastomotic leakage [3] and its clinical consequences [3–6]. The morbidity associated with a temporary defunctioning stoma is, however, considerable with complications such as dehydration and renal failure [4, 7–9] as well as parastomal hernia and skin irritation and ulceration [7]. Defunctioning stomas are usually reversed approximately three months after formation but it is not unusual that the stoma is left in place much longer and for some patients it becomes permanent [10].

Hospital costs associated with the treatment of rectal cancer are considerable, and the formation of a temporary stoma accounts for additional costs, including potential readmissions and a second operation (stoma closure) [11, 12]. Early closure of a temporary stoma has been associated with significantly fewer postoperative complications up to 12 months after rectal resection in a randomized controlled trial [13]. Although the routine use of a temporary stoma in patients operated with low anterior resection increases the hospital costs of surgical treatment for rectal cancer [12], early closure of such may be a cost-effective alternative [14].

The aim of the present study was to perform a cost analysis 12 months after rectal resection for cancer in a multicentre randomized trial comparing early vs. late closure of a temporary ileostomy (EASY trial) [15].

## Materials and methods

### EASY trial

The EASY trial was designed as a randomized multicentre controlled trial [15] comparing early with late closure of a temporary ileostomy after rectal resection. The primary endpoint was the mean number of complications after rectal resection and a secondary endpoint included health-related quality of life. Both results have been published previously [13, 16]. Inclusion of participants was made after rectal resection (TME for rectal cancer including the creation of a temporary ileostomy). Exclusion criteria were ongoing steroid treatment, diabetes mellitus, signs of postoperative complications and inability to understand Danish or Swedish. Patients with no adverse events were invited to participate. After informed consent, the patients underwent investigation with a contrast computed tomography scan (CT scan) and/or a flexible endoscopy of the rectum to confirm the integrity of the anastomosis. This was performed 6 to 8 days after index operation with stoma creation. Patients were then randomized to either the intervention group with early closure (8–13 days after stoma creation) or to the control group with late closure (> 12 weeks after stoma creation) of the temporary ileostomy. Eight Danish and Swedish hospitals participated in the trial during February 2011 to November 2015. Three centres (with a total number of 8 patients) were excluded due to failure of maintaining a screening log.

The present study comprised a secondary endpoint and aimed at comparing the costs between the two groups within 12 months after the rectal resection (stoma formation).

### Health economic methodology

Consenting patients were followed up at stoma closure and at 3, 6 and 12 months after rectal resection regarding postoperative complications and health-related quality of life (primary and secondary endpoints). The resource use analysis was carried out at 12 months. All data were collected through case report forms (CRF). For the analysis, unit costs were derived from Swedish sources and applied for all patients. The analysis included costs accumulated during 12 months after stoma formation (rectal resection).

### Resource use

For all surgical procedures, including rectal resection and stoma closure as well as reoperations, unit costs were derived from the Swedish Association of Local Authorities and Regions (SALAR, Sveriges kommuner och landsting, http://www.skl.se), which is based on approximately 85% of all inpatient procedures in Sweden. Readmissions without surgical interventions were calculated based on the actual days of hospital admission in a regular ward. Prior to analysis, since only one patient (control group) required admission to the intensive care unit (ICU), the decision was made not to include this in the cost calculation, as previously suggested [17]. Hence, there would be a risk of adding a rare and costly event that single-handedly could tip the results in one direction. Number of days with a temporary ileostomy (and for a few patients, permanent colostomy) was registered. Because of the fluctuation of stoma function, the resource items needed per day for stoma care were estimated in collaboration with a specialized stoma nurse for a typical functioning stoma.

Only outpatient radiological and endoscopic examinations were registered in the CRFs. Radiological examinations that served as 12 months oncological evaluation after rectal cancer surgery (as part of regular follow-up) were not included in the analysis, as these did not differ between the two groups. The CT scan and/or flexible endoscopy of the rectum that was performed prior to randomization was not included in the cost analysis, as this was a procedure solely for study inclusion and did not differ between the two groups.

All outpatient visits (outpatient clinic nurse, stoma nurse and surgeon) were registered and included in the cost analysis. The cost of chemotherapy was not included in the analysis as the distribution was equal between the two groups and was independent of timing of closure of the temporary ileostomy. Indirect costs such as sick leave were not included, as registry data were not available for the whole study population.

### Unit costs

Surgical procedure codes (classified using the Nordic Medico-Statistical Committee ‘NOMESCO’ Classification of Surgical Procedures version 1.16) were retrieved from the national cost per patient database for the year 2016 from SALAR where every surgical procedure has a mean cost representing one hospital admission. These data were used for the calculation of all inpatient surgical procedures, including rectal resection, stoma closure and reoperations. Costs for outpatient procedures (surgical intervention and endoscopy) were derived from the outpatient Diagnosis Related Groups (DRG). Stoma material costs were obtained from pharmacy retail prices in Sweden. All costs were adjusted to the price year 2016 and converted from Swedish crowns (SEK) to United States dollar (USD) according to the purchasing power parities (PPP) for the gross domestic product (Organisation for Economic Co-operation and Development, http://www.oecd.org).

### Trial registration

The protocol was registered at http://www.clinicaltrials.gov (NCT01287637) prior to patient inclusion.

### Randomization

Consenting patients who fulfilled the inclusion criteria were randomized either to the intervention group with early closure (day 8–13 after stoma creation) or to the control group with late closure (> 12 weeks after stoma creation) of the ileostomy. Randomization was executed in computer-generated blocks of six. The randomization was performed in the surgical ward using sequentially numbered, thick, opaque and sealed envelopes. Blinding of the intervention was not possible.

### Statistical analysis

The present study was part of a randomized controlled trial with power calculated for the primary endpoint (postoperative complications up to 12 months). The group size in EASY was set to 72 patients per group [13]. Since the health-related quality of life analysis was unable to detect a difference between the two groups [16], this variable was not included in the health economic analysis. As the intervention resulted in less morbidity [13], and is presumably less costly, the study was considered superior in terms of effectiveness and we did not plan for calculation of quality-adjusted life years (QALYs). Complete data were available for 106 patients (95%). In order to enable total cost calculation, missing values of specific cost components were imputed with the mean for the whole cohort. The six patients with missing data represented both the intervention and control group (*n* = 3 in each), and there were no differences in postoperative complications (one patients in each group had a grade IIIa complication according to Clavien–Dindo classification of complications). For the analysis and clinical outcomes of interest, descriptive statistics were used. Two-sample t test was used for the comparison of mean costs between the two groups. Due to skewed data and to assess robustness, a non-parametric bootstrap analysis was performed, as recommended [18]. P values smaller than 0.05 were considered statistically significant. A supporting analysis was performed, adjusting for sex, age, BMI, comorbidity and radiation in a regression model (in accordance with previous adjusted analysis for the primary endpoint). The software packages SPSS® 23 software (IBM, Armonk, New York, USA) SAS 9.4 (SAS Institute, Cary NC) and R 3.2.3 software [19] were used for statistical analysis.

## Results

**Fig. 1:**
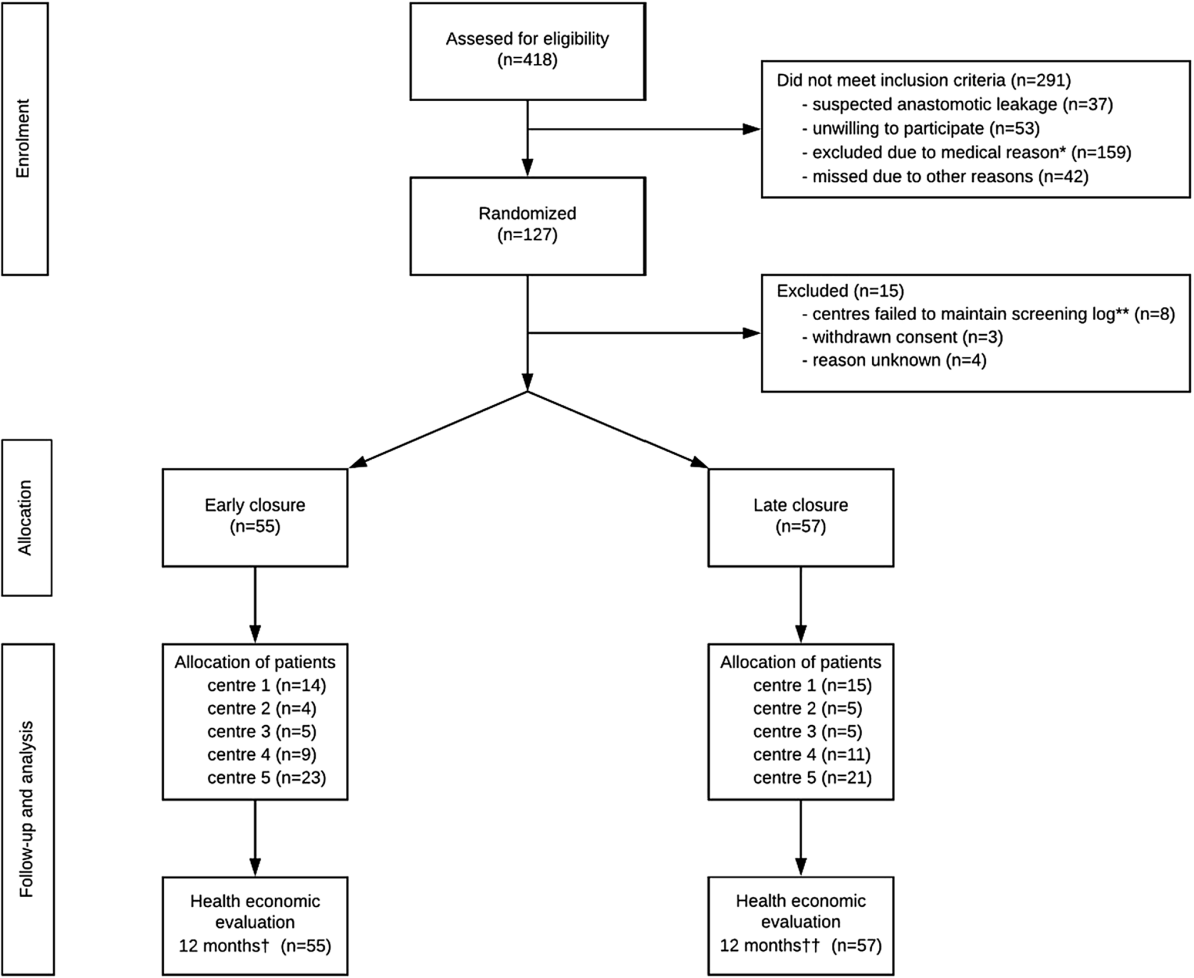
Study flow chart. *Excluded after clinical evaluation (*n* = 91), diabetes (*n* = 28), permanent or no stoma (*n* = 29), steroid treatment (*n* = 3), other (*n* = 8). **Centre 6 (*n* = 2), centre 7 (*n* = 3) and centre 8 (*n* = 3). ^†^One patient died within 12 months (237 days). ^††^One patient died within 12 months (294 days). Figure has previously published in Danielsen et al. [12] and Park et al. [16]

The EASY trial enrolled 112 patients between February 2011 and November 2014. The last follow-up was in November 2015. There were 55 patients in the early closure group (intervention) and 57 patients in the late closure group (control) (Fig. 1). One patient in each group died within 12 months after rectal resection and the costs for these patients were included in the analysis (Fig. 1). There were no violations of the randomization. Baseline demographic characteristics including comorbidity, cancer stage, chemo- and radiotherapy and body mass index (BMI) did not differ between the two groups, but there was a larger female population in the early closure group (Table 1). As reported previously [13], there were significantly fewer complications in the early closure group at 12 months after rectal resection. There was no difference in more severe complications (Clavien–Dindo grade IIIb and higher) (Table 1). The median time with a temporary ileostomy was 11 and 148 days in the early and late closure group, respectively. The total length of hospital stay for the rectal resection and the loop ileostomy closure (either as one or two admissions depending on treatment group) did not differ between the groups, but there were more readmissions in the late closure group (Table 1). The resource variables, unit costs and corresponding sources are listed in Table 2. Mean cost per patient and difference in resource use variables at 12 months are shown in Table 3. All resource use variables were more costly in the late closure group, except for ileostomy closure. This was due to the fact that more patients in the early closure group (25 patients compared to 22 patients in the late closure group) underwent a small bowel resection at closure (not requiring laparotomy), which resulted in a cost difference. The total difference in mean cost per patient was 4060 USD ($) in favour of early closure (95% confidence interval 1121; 6999, *p* value <0.01). A non-parametric bootstrap based on 2000 iterations showed similar results (Table 4). The adjusted analysis did not alter the results (Table 4). The predominant cost affecting factors were reoperations, readmissions and endoscopic examinations. The number of endoscopic examinations was in a sense protocol driven, to the extent that patients in the late closure group often undergo an additional endoscopic examination in order to confirm the integrity of the anastomosis, prior to stoma closure. A sensitivity analysis was therefore performed where the cost of an additional flexible sigmoidoscopy was added to early closure group and the patients in the late closure group that had not undergone any endoscopic examinations within 12 months after rectal resection. This analysis resulted in an overall difference in mean cost per patient of $3608, in favour of early closure (95% confidence interval 668; 6549, *p* value 0.02).

**Table 1:** Baseline characteristics of included patients

	Randomization group	
	Early closure (*n* = 55)	Late closure (*n* = 57)
Age (years)	67 (36–82)	67 (39–81)
Sex (female/male)	31 (56%)/24 (44%)	21 (37%)/36 (63%)
Body mass index (kg/m^2^)	24 (17–32)	23 (19–35)
Comorbidity (number of patients)	23 (42%)	24 (42%)^a^
Ischaemic heart disease	5	8
Hypertension	17	13
Chronic obstructive pulmonary disease (COPD)	2	2
Renal disease	0	0
Other	9^b^	4^c^
Smoking	6 (11%)	4 (7%)
If yes, number of pack years (years)^d^	30 (16–30)	26 (20–50)
Alcohol use (> 60 g of alcohol a day)	0	0^e^
Employed/unemployed	25 (45%) / 29 (53%)^e^	25 (44%) / 32 (56%)
Radiotherapy	16 (29%)	16 (28%)
If yes, long-term radiotherapy	5 (9%)	5 (9%)
Adjuvant chemotherapy	22 (40%)	23 (40%)
Clinical stage according to “The Union for International Cancer Control” (UICC)	54/55^f^	53/57^g^
I	12 (22%)	19 (33%)
II	21 (38%)	13 (23%)
III	18 (33%)	20 (35%)
IV	3 (5%)	1 (2%)
Method of evaluation of anastomosis prior to ileostomy closure		
CT scan	14 (25%)	19 (33%)
Flexible endoscopy of the rectum	14 (25%)	10 (18%)
CT scan and flexible endoscopy	27 (49%)	28 (49%)
Total length of hospital stay for rectal resection and loop ileostomy closure (days)	14 (11–42)	14 (7–44)
Total number of complications at 12 months after rectal resection. Classification according to Clavien–Dindo		
Grade I–II	27	56
Grade IIIa	6	19
Grade IIIb	7	5
Grade IVa	1	2
Grade IVb–V	0	0
Readmission	13 (24%)^e^	28 (49%)

Data previously published: Danielsen et al. [13]

Numbers are in median. Percentages or range is given in parenthesis

^a^Missing (*n* = 2)

^b^Asthma (*n* = 2), depression (*n* = 1), idiopathic thrombocytopenic purpura (ITP) (*n* = 1), lymphoma (*n* = 1), Waldenström macroglobulinemia (*n* = 1), osteoporosis (*n* = 1), Sjögren syndrome (*n* = 1), thyrotoxicosis (*n* = 1)

^c^Depression (n = 1), hyperlipidaemia (n = 1), hypothyreosis (n = 1), meningioblastoma (n = 1)

^d^Missing intervention group (n = 3) and control group (n = 1)

^e^Missing (*n* = 1)

^f^Missing (*n* = 1)

^g^Missing (*n* = 1) and three patients had T0N0M0 and therefore are not classified

**Table 2:** Unit cost per resource use variable

Resource variables	Unit cost (USD)^a^	Unit	Source
Rectal resection with temporary loop ileostomy^b^	19911	Per procedure	Swedish Association of Local Authorities and Regions
Stoma closure with bowel resection^b^	8838	Per procedure	Swedish Association of Local Authorities and Regions
Stoma closure without bowel resection^b^	7651	Per procedure	Swedish Association of Local Authorities and Regions
Reoperation^b^	3576–33,350	Per type of reoperation	Swedish Association of Local Authorities and Regions
Length of hospital stay—readmission	693	Per day	Sahlgrenska University Hospital, Gothenburg, Sweden
Outpatient visits	77–80	Per visit	Sahlgrenska University Hospital, Gothenburg, Sweden
Outpatient endoscopy	694–924	Per examination	Sahlgrenska University Hospital, Gothenburg, Sweden
Outpatient radiology	77–1713	Per examination	Sahlgrenska University Hospital, Gothenburg, Sweden
Stoma appliances	8	Per day with ileostomy	Pharmacy retail price
Stoma appliances	13	Per day with colostomy	Pharmacy retail price

^a^Exchange rate according to prices and purchasing power parities (PPP) 2016

^b^Includes the mean of all costs associated with a certain surgical procedure (rectal resection, loop ileostomy closure with/without bowel resection and different reoperations)

**Table 3:** Mean cost per patient and difference in mean cost per resource use variable

	Early closure (USD)	Late closure (USD)	Difference early–late (USD)
Index Surgery	19,911	19,911	0
Loop ileostomy closure^b^	8191	7854	337
Readmission with reoperation	1215	1637	− 422
Readmission without reoperation	873	2309	− 1436
Temporary ileostomy—stoma appliances	113	1437	− 1324
Permanent colostomy—stoma appliances	0	39	− 39
Outpatient visits^a^	141	536	− 396
Stoma care nurse	*7*	*241*	
Surgeon	*122*	*303*	
Outpatient endoscopy	521	964	− 443
Outpatient radiology^a^	279	615	− 337
Regular X-ray	*18*	*57*	
Ultrasound	*24*	*54*	
Computer tomography scan	*163*	*409*	
Magnetic resonance imaging	*70*	*98*	
Total cost at 12 months	31,243	35,303	− 4060

Missing data are replaced by imputed values which are derived from the mean cost per resource use variable for the whole cohort

^a^Subcategories (italics) are presented as mean costs per patient

^b^Three patients did not undergo closure within 12 months (closure > 365 days *n* = 2, deceased within 12 months n = 1)

**Table 4:** Mean total cost and difference in mean total cost per patient

	Early closure (USD)	Late closure (USD)	Difference early–late (USD)	SE	95% CI	*p* value
Total cost at 12 months^a^	31,243	35,303	− 4060	1483	− 6999, − 1121	0.007*
Non-parametric bootstrap^b^				1426	− 7014, − 1425	0.004*
Adjusted analysis^c^	31,214	35,387	− 4173	1523	− 7194, − 1153	0.007*

Values are in mean

^a^Normal *t* test

^b^Non-parametric bootstrap based on 2000 iterations

^c^Adjusted for sex, BMI, comorbidity, age, radiation

*Statistically significant

## Discussion

The results showed that early closure of a temporary ileostomy after rectal resection for cancer was less costly than late closure (standard care) in a randomized study setting. Early closure was associated with less use of outpatient resources (outpatient visits, procedures and diagnostic investigations) as well as fewer readmissions.

Readmission is a known factor affecting total direct healthcare costs [20] and previous studies have identified that the presence of a stoma has been associated with increased readmission rates within 30 to 90 days after colorectal surgery [20, 21]. One study reported an overall 90-day readmission rate of 23% [21]. In the present study, readmissions were twice as common in the late closure group (49%), compared to early closure (24%). This includes all readmissions within 12 months after rectal resection but excludes the readmission required for stoma closure.

A randomized trial of 234 patients found that the use of a defunctioning loop stoma reduced the rate of symptomatic anastomotic leakage after rectal resection for cancer [3]. A recently published five year analysis of the costs in the same study showed that the use of a defunctioning stoma was more expensive due to the need for a second operation (closure) and costs for stoma appliances, despite the cost savings associated with reduced frequency of symptomatic anastomotic leakage [12]. Since previous studies indicated benefits from the use of a defunctioning stoma [3, 5], the standard surgical treatment has included the formation of such. The EASY trial showed that early closure of a temporary ileostomy in selected patients without clinical or radiological signs of anastomotic leakage was associated with significantly fewer postoperative complications up to 12 months after rectal resection. Even though risk factors for anastomotic leakage after rectal resection for cancer are known, we still lack the tools to preoperatively identify which patients truly need a temporary stoma [22, 23]. The clinical postoperative evaluation and inclusion process used in the EASY trial, followed by early closure might therefore be a cost-effective and safe alternative, bearing in mind that early closure is only an option for patients without any signs of postoperative complications. In the trial, evaluation of the anastomosis was performed on postoperative day 6–8, and patients randomized to the intervention group underwent early closure 8–13 days after rectal resection. The total length of hospital stay (for rectal resection and loop ileostomy closure) did not differ between the groups. Patients in the trial may have had an overall longer hospital stay compared to patients undergoing rectal resection for cancer, outside the trial, due to the clinical evaluation and anastomotic investigation. However, given the potential risks of including patients with an anastomotic leak, this was considered necessary from a safety aspect. The present study revealed that the late closure group underwent more endoscopic examinations. This is probably partly due to the protocol, in the sense that several patients in the late closure group underwent an extra examination prior to closure of the stoma, since the previous examination would have been performed between three and eight months earlier. A sensitivity analysis was therefore performed in order to compensate for the extra flexible endoscopy of the rectum that would ‘burden’ the control group in this case. In this analysis, we could see that it had little effect on the overall expense, and the significant cost difference between the two groups remained. One patient in the trial required admission to the intensive care unit. This was a rare and costly event, and based on previous recommendations [17], it was not included in the cost analysis. Since this patient belonged to the late closure group, we may have consequently underestimated the true difference between the groups. However, this did not change the conclusion of the study.

The strength of the present study includes the study design (randomized controlled trial). The robust results with regard to clinical effectiveness, significant differences in resource use and costs, high rate of follow-up and few missing values together with performed sensitivity analyses suggest high internal validity. The inclusion of only direct costs (for example excluding sick leave) and the fact that the study was not powered for the present outcome are identified as limitations.

## Conclusion

The significant cost reduction in this study, together with results of safety and efficacy from the randomized controlled trial, supports the routine use of early closure of a temporary ileostomy after rectal resection for cancer in selected patients without signs of anastomotic leakage.
